# Chaotic time series prediction for prenatal exposure to polychlorinated biphenyls in umbilical cord blood using the least squares SEATR model

**DOI:** 10.1038/srep25005

**Published:** 2016-04-27

**Authors:** Xijin Xu, Qian Tang, Haiyue Xia, Yuling Zhang, Weiqiu Li, Xia Huo

**Affiliations:** 1 Laboratory of Environmental Medicine and Developmental Toxicology, and Provincial Key Laboratory of Infectious Diseases and Molecular Immunopathology, Shantou University Medical College, Shantou 515041, Guangdong, China; 2 Department of Cell Biology and Genetics, Shantou University Medical College, Shantou 515041, Guangdong, China; 3 School of Environment, Guangzhou Key Laboratory of Environmental Exposure and Health, Guangdong Key Laboratory of Environmental Pollution and Health, Jinan University, Guangzhou 510632, Guangdong, China

## Abstract

Chaotic time series prediction based on nonlinear systems showed a superior performance in prediction field. We studied prenatal exposure to polychlorinated biphenyls (PCBs) by chaotic time series prediction using the least squares self-exciting threshold autoregressive (SEATR) model in umbilical cord blood in an electronic waste (e-waste) contaminated area. The specific prediction steps basing on the proposal methods for prenatal PCB exposure were put forward, and the proposed scheme’s validity was further verified by numerical simulation experiments. Experiment results show: 1) seven kinds of PCB congeners negatively correlate with five different indices for birth status: newborn weight, height, gestational age, Apgar score and anogenital distance; 2) prenatal PCB exposed group at greater risks compared to the reference group; 3) PCBs increasingly accumulated with time in newborns; and 4) the possibility of newborns suffering from related diseases in the future was greater. The desirable numerical simulation experiments results demonstrated the feasibility of applying mathematical model in the environmental toxicology field.

Polychlorinated biphenyls (PCBs) belong to the family of persistent organic pollutants on the basis of persistence and bioaccumulation, and causing serious harm to humans and experimental animals^1^,^2^,^3^. PCBs were widely used in industrial products since the 1970s, and can be released into the environment in the recycling of transformer and capacitor, and have become ubiquitous environmental pollutants^4^. PCBs can be measured from the Arctic sea dog and Galapagos yellowfin to the Antarctic bird egg^5^,^6^, as well as in human breast milk^7^,^8^,^9^,^10^,^11^,^12^. As exogenous chemical substances, PCBs have been reported to bring about various adverse effects on reproduction, nerve and immune systems, particularly in the development and growth of newborns^13^,^14^. For example, PCBs can cause serious effects on the neurobehavior of newborns and children, including growth retardation, cognition impairment, clumsiness, and lower IQ^15^,^16^,^17^,^18^,^19^,^20^,^21^,^22^.

Guiyu is a global center for electronic waste (e-waste) recycling and processing, with a 30-year history of e-waste dismantling industry involving 60–80% of households. In order to save costs, Guiyu’s family workshops recycle e-waste by adopting the most direct and primitive methods, including simple manual dismantling, burning of plates, acid pickling, and open burning, causing severe pollution to the local environment^23^. In addition, PCBs are widely used as insulating oil for transformers and capacitors, heat carriers, and lubrication, and can be released into the environment during the disassembly of e-waste^24^. Research has shown that the PCB concentrations in Guiyu air amounts to 414.8 ng/m^3^, 88-fold higher than the surrounding area (4.7 ng/m^3^), leading to PCB exposure of local residents through inhalation^25^. PCBs have also been detected in fish^26^. Based on the ability of PCBs to accumulate in the human body, these results indicate that local residents will have high somatic PCB levels due to prolonged PCB exposure from recycling e-waste, potentially bringing severe harm to their health, especially to pregnant women and children^27^,^28^,^29^,^30^,^31^,^32^,^33^.

Chaos is a form of motion that is due to non-linear dynamics widely existing in nature^34^. A chaotic time series is a univariate time series manufactured by observing and sampling a chaotic system^35^. In a chaotic system, dynamic behavior of the overall system is investigated by a time series that forecasts the chaos sequence. Because data in environmental toxicology are chaotic, a chaotic time series is relevant to environmental toxicology. At present, chaotic time series prediction has been widely focused and applied to the economy, signal processing, and automatic control^36^,^37^,^38^,^39^,^40^,^41^,^42^,^43^. However, few studies have applied chaotic time series analysis to environmental medicine and developmental toxicology.

In this study, based on a least squares SEATR model, we designed special steps for chaotic time series prediction targeting 262 serum samples of umbilical cord blood from the e-waste dismantling district of Guiyu using Haojiang as a reference area, to achieve a chaotic time series prediction for prenatal PCB exposure. The forenamed scheme’s validity was further verified by numerical simulation.

## Materials and Methods

### Data source

A total of 262 umbilical cord blood samples were collected, 189 from Guiyu (exposed group) and 73 from Haojiang (reference group), between September 2010 and September 2011. This research received approval from the Ethics Committee of Shantou University Medical College. Informed consent forms have been obtained from all subjects and methods were carried out in accordance with the approved guidelines.

### Methods

Samples were analyzed with an Agilent 7890A gas chromatograph coupled with an Agilent 5975C mass spectrometer (Agilent Technologies, USA). Using 1 ml umbilical cord blood serum from each subject, 7 PCB congeners (PCB-28, PCB-52, PCB-101, PCB-138, PCB-153, PCB-180 and PCB-209) were measured simultaneously, and expressed as ng/g lipid. The proposed method was established based on the Chaos theory and SETAR model theory. MATLAB software version R2008a was used for the statistical analysis, and Adobe Photoshop CS6 software was used for processing figures.

### Design of prediction steps

A total of 131 of 189 samples from the exposed group multiplied by 7 PCB congeners of every newborn would result in 917 samples to constitute time series 1 in which the minimum embedding dimension was 1, time delay was 1, and average period was 7. A total of 63 of 73 samples formed the reference group multiplied by 7 PCB congeners of every newborn to result in 441 samples to constitute time series 2 in which the minimum embedding dimension was 1, time delay was 1, and average period was 7.

A total of 130 out of 189 samples from the exposed group multiplied by the 5 conditions of newborn birth, which included newborn weight, height, gestational age, Apgar score and anogenital distance, the units of which were respectively kg, cm, week, score and mm, would shape 650 samples to constitute time series 3, in which the minimum embedding dimension was 1, time delay was 1, and average period was 5. Similarly, a total of 49 out of 73 samples fetched from the reference group multiplied by 5 would shape 245 samples to constitute time series 4, in which the minimum embedding dimension was 1, time delay was 1, and average period was 5.

The largest Lyapunov exponent of forenamed time series was computed by the Wolf arithmetic^44^, and the largest Lyapunov exponent of time series 1 to 4 were 2.8694, 2.4562, 1.6552 and 1.4174, respectively.

The least squares SEATR model was utilized to predict a chaotic time series in the exposed group. SEATR (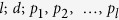) is the simplest nonlinear time-series model, put forward by H. Tong in 1978, the general type of which is


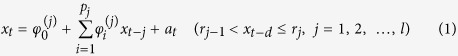


In the above formula, *l* is the number of the threshold segment that is divided by the magnitude of 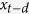; 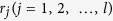 is the threshold value dividing the threshold segment; *d* is the delay step; 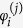 and 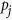 respectively are the parameter and the order of the model in the 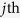 threshold segment.

The difference between the SEATR model and AR model is that 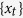 is distributed into the different interzone segments (threshold segments) by the magnitude of 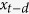 and the different AR model is adopted to be described in the different section. The AR model is a particular case of the SEATR model in which *l* = 1 and *d* = 0.

The least squares SEATR model is a modeling approach that was suggested for a chaotic time series having seasonal fluctuations. By analyzing the sequential value of the same time in the period of every season, this model extracts the tendency of the season by aiming at the change of the internal sequence value of the period of every season and extracting the unseasonal ingredient. This establishes the most accurate model.

The specific steps were as follows:

Invoke the least squares method to compute the comprehensive seasonal parameters;

Compute the seasonal items;

Estimate the parameters of the SEATR model by the least squares method;

Fit the stochastic term to the SEATR model;

Forecast the trend term;

Forecast the seasonal items;

Forecast the stochastic term;

Forecast the result;

Error list;

Error sum;

Forecast average error;

Forecast the length;

Forecast the data;

Obtain temporary data;

Forecast the stochastic term by the SEATR model;

Add new data to temporary data to forecast;

Forecast the trend term;

Forecast the seasonal items;

Forecast the stochastic term;

Forecast the result;

Provide a figure of the comparison of the predicting outcomes and the original.

### Numerical simulation

In order to evaluate the proposed model, 917 and 441 samples of the chaotic time series for the exposed group, including the 7 kinds of PCB congeners, were analyzed. Figure S1 and S2 show the power spectrum of chaotic time series in the exposed group and the reference group, respectively. The power spectrum of time series is continuous but not smooth, with occasional noise background, indicating both time series possesses complicated non-linear chaos characteristics. Figure S3 and S4 exhibit the relative error (RE) trend of the chaotic time series in the exposed and reference groups, which demonstrate that the fitting data and the original data are anastomosed better, and the average forecast error being 0.5443 and 0.6143, respectively. We further compared the prediction and the original chaotic time series between the exposed group and the reference group (Figure S5 and S6). The results show that the short-term prediction can be extended to the future, with a prediction length was 50 days. The prediction part of the graph predicts PCBs to increasingly accumulate in newborns with time at a greater rate, as well as indicates the enhanced possibility of newborns suffering from related diseases in the future.

## Discussion

Chaotic time series prediction based on nonlinear systems has become a hot spot, which was widely applied for the traffic network, earthquake prediction, and weather forecasting^45^,^46^,^47^, and many interesting results have been provided by several researchers in recent years^48^,^49^. This study attempts to formulate a chaotic time series prediction based on the least squares SEATR model to explore the relationship between prenatal PCB exposure in umbilical cord blood and neonatal criteria at birth in an e-waste dismantling area.

In the model, optimal delay time (*τ*) and embedding dimension (*m*) in the phase space were selected, and the largest Lyapunov exponent was computed by the Wolf arithmetic^44^. If the largest Lyapunov exponent of a time series is greater than 0, the time series will be chaotic; Similarly, when the largest Lyapunov exponents of the chaotic time series of the exposed groups are greater than that from the reference groups, it would suggests the possibility of suffering from related disease for newborns in the exposed group was greater than the reference group; In the present study, the largest Lyapunov exponents in each of the two exposed groups, including 7 PCB congeners and 5 indicators of birth status, were greater than 0, and the largest Lyapunov exponents of the two corresponding reference groups show that there is a close relationship between the 7 PCB congeners and the 5 birth status indicators in both the exposed and reference groups, and that the largest Lyapunov exponent of the exposed group, including 5 birth status indicators was greater than that in the reference group, indicating that the 7 PCB congeners in the exposed group correlate with the 5 birth status indices. This would equate to exposed newborns being greater risks than for the reference newborns.

We utilize the least squares SEATR model to predict a chaotic time series in the exposed group, and established accurate model with the specific steps listed. Chaotic time series prediction based on nonlinear systems shows in general superior performance over the traditional statistical fitting methods. The traditional statistical fitting methods, such as autoregressive (AR), moving average (MA), and autoregressive moving average (ARMA) models, have been used in chaotic time series prediction^50^. However, due to the inherent linearity assumptions, the above conventional mathematical tools are not well suited for dealing with ill-defined and uncertain systems. The SEATR model is a nonlinear prediction approach and fits for real systems to be described and forecast, and especially suitable for long term forecast. Therefore, the proposal scheme was suitable for analyzing prenatal PCB exposure due to chaotic characteristic of data in environmental toxicology.

Furthermore, we conduct a numerical simulation using time series 1and time series 2 to verify the performance of the model. The power spectrum of the proposed methods is continuous but not smooth, with occasional noise background, indicating the time series possesses complicated non-linear chaos characteristics. The relative error and the power spectrum of prediction are also shown. In addition, we provide a figure of the comparison of the predicting outcomes and the original. Through multiple matching and the forecast generated by the proposed model we conclude that the short-term prediction could be extended to the future with a higher accuracy and a smaller relative error in the prediction, demonstrating the fitting data and the original data are anastomosed better, further verifying the applicability of the proposed model.

Although this is the first time that chaotic time series has been applied to analyze environmental toxicology data and to predict the trend of newborn PCB exposure with better accuracy, the proposal model has its limitations. We are unable to compare with other forecast models or methods such as the grey forecast or neural network prediction due to the fact that they are rarely utilized in medicine-related or environmental context research. The results of the estimating indices such as Square Error (SE), Mean Square Error (MSE), and Sum of squared errors (SSE) between the proposed method and other prediction models should be listed to validate its performance. Therefore, the applicability and superiority of the proposed model requires further validation involving other environmental data.

## Conclusion

This study uses PCB concentrations in the umbilical cord blood of newborns, from individuals residing in an e-waste contaminated area, and describes a chaotic time series prediction for prenatal PCB exposure. The biological significance of the proposed method lies in that seven kinds of PCB congeners influence five kinds of birth status indices, and can forecast the long term greater risks of newborns exposed to PCBs compared to the reference group. Our results further predict PCBs will increasingly accumulate in newborns with time, as well as strengthen the possibility of newborns suffering from related diseases in the future.

## Additional Information

**How to cite this article**: Xu, X. *et al*. Chaotic time series prediction for prenatal exposure to polychlorinated biphenyls in umbilical cord blood using the least squares SEATR model. *Sci. Rep*. **6**, 25005; doi: 10.1038/srep25005 (2016).

## Supplementary Material

Supplementary Information
