# Supplementary material for: Chaotic time series prediction for prenatal exposure to polychlorinated biphenyls in umbilical cord blood using the least squares SEATR model
Source: Sci Rep. 2016 Apr 27;6:25005. doi: 10.1038/srep25005 (PMC4846991; doi:10.1038/srep25005)
Supplement: Supplementary Information [file srep25005-s1.pdf]

Chaotic time series prediction for prenatal exposure to polychlorinated biphenyls in umbilical cord blood using the least squares SEATR model

Xijin Xu, Qian Tang, Haiyue Xia, Yuling Zhang, Weiqiu Li, Xia Huo

Appendix

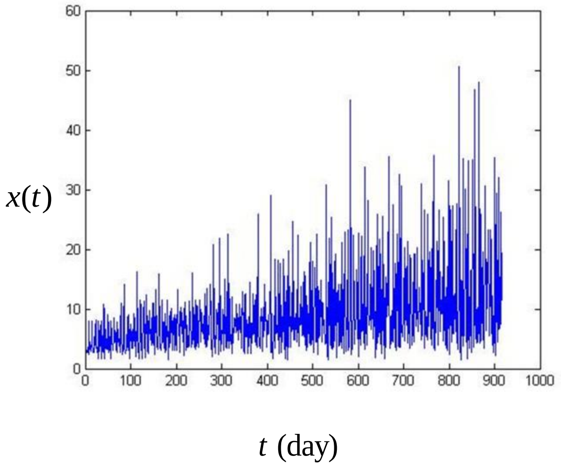

Figures S1. Chaotic time series in the exposed group.

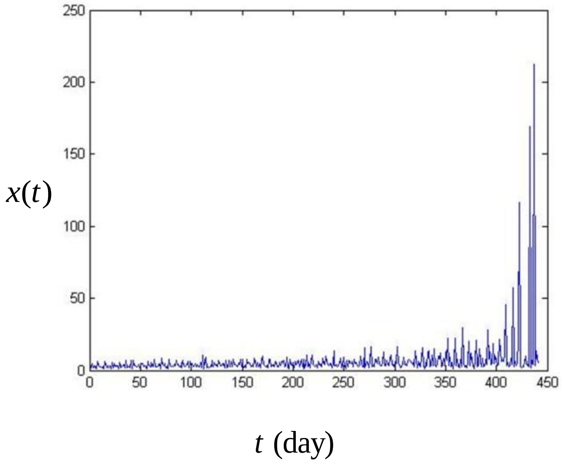

Figures S2. Chaotic time series in the reference group.

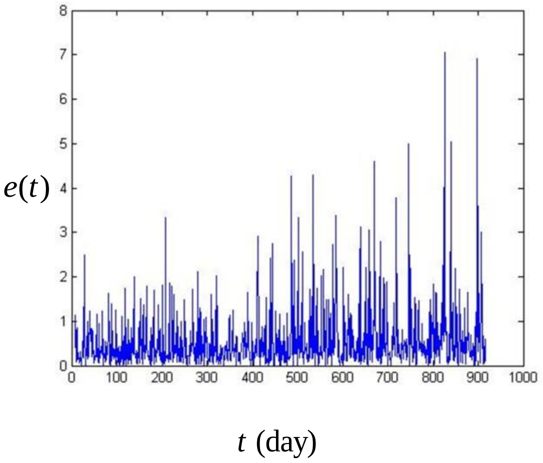

Figures S3. Relative error trend of the chaotic time series in the exposed group.

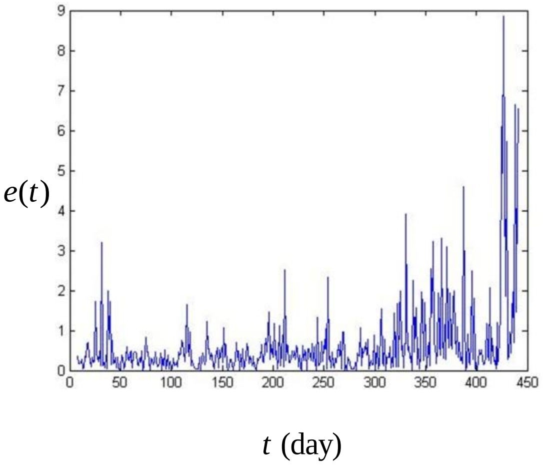

Figures S4. Relative error trend of the chaotic time series in the reference group.

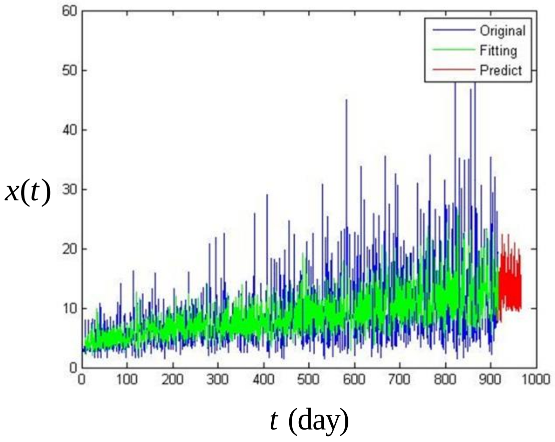

Figures S5. Comparison of the exposed group’s prediction and the original chaotic time series.

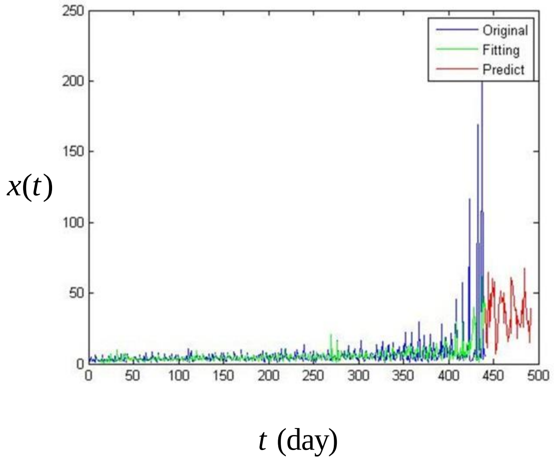

Figures S6. Comparison of the reference group’s prediction and the original chaotic time series.
